# Changing summer precipitation variability in the Alpine region: on the role of scale dependent atmospheric drivers

**DOI:** 10.1007/s00382-021-05753-5

**Published:** 2021-04-10

**Authors:** Klaus Haslinger, Michael Hofstätter, Wolfgang Schöner, Günter Blöschl

**Affiliations:** 1 Climate Research Department, Central Institute for Meteorology and Geodynamics (ZAMG), Hohe Warte 38, 1190 Vienna, Austria; 2 Department for Environment and Energy Affairs, Federal Government of Lower Austria, Landhausplatz 1, 3109 St. Pölten, Austria; 3 Department of Geography and Regional Sciences, University of Graz, Heinrichstraße 36, 8010 Graz, Austria; 4 Institute for Hydraulic and Water Resources Engineering, and Centre for Water Resource Systems, Vienna University of Technology, Karlsplatz 13, 1040 Vienna, Austria

## Abstract

Summer precipitation totals in the Alpine Region do not exhibit a systematic trend over the last 120 years. However, we find significant low frequency periodicity of interannual variability which occurs in synchronization with a dominant two-phase state of the atmospheric circulation over the Alps. Enhanced meridional flow increases precipitation variability through positive soil moisture precipitation feedbacks on the regional scale, whereas enhanced zonal flow results in less variability through constant moisture flow from the Atlantic and suppressed feedbacks with the land surface. The dominant state of the atmospheric circulation over the Alps in these periods appears to be steered by zonal sea surface temperature gradients in the mid-latitude North Atlantic. The strength and the location of the westerlies in the mid-latitude Atlantic play an important role in the physical mechanisms linking atmosphere and oceanic temperature gradients and the meridional/zonal circulation characteristics.

## Introduction

The Alps are often referred to as Europe’s water tower because of their immense water resources (European Environment Agency 2009) provided also to downstream areas. Annual precipitation over the Alpine region averaged around 1100 mm during the late twentieth century (Frei and Schär 1998). Summer is the wet season, except for the very southern parts of the region.

While decadal scale changes in mean summer precipitation have been investigated in detail (Anders et al. 2014), the variability of summer precipitation between years has received relatively little attention (Pendergrass et al. 2017; Yin and Roderick 2019). Yet increases in the inter-annual variability of precipitation would imply an increasing probability of hydrometeorological extremes and thus the occurrence of flood and drought events (Merz and Blöschl 2003). It is therefore important to understand the changes and potential drivers of interannual variability of summer precipitation on multidecadal time scales.

Zveryaev (2004) investigated the interannual variability of both winter and summer precipitation over Europe. He found the leading modes of variability for summer to be related to the North Atlantic Oscillation (NAO), although Zveryaev and Allan (2010) highlights the role of local processes (e.g. evapotranspiration), in periods when the large scale circulation is weak. The NAO seems to be important in general terms for the European climate, but it is less so for the Alpine Region and in the summer season in particular (Haslinger et al. 2019). In another study Zveryaev et al. (2016) analyzed the interannual variability of the hydrological cycle in summer for selected regions in Europe. Their results highlight the importance of atmospheric moisture transport into a specific region as a driver for the interannual variability of summer precipitation. They found that the coupling between soil moisture and precipitation is of particular relevance for Central Europe. Similar results have been obtained in the recent study of Haslinger et al. (2019) who showed that soil moisture precipitation feedbacks are very important for drought development over the Alpine Region.

Most of the literature cited above has analyzed the interannual variability of precipitation by identifying associated leading modes of variability of the atmospheric circulation. In contrast multidecadal variability was generally ignored, despite it is still not clear if recent changes of observed precipitation are due to internal variability or a consequence of external climate forcing. Interestingly, changes in the mean state of climatic variables (e.g. temperature, precipitation) on multidecadal time scales are more often considered, with various authors investigated the driving role of the Atlantic Multidecadal Oscillation, (AMO, Enfield et al. 2001). As these variations of average sea surface temperatures in the North Atlantic are not true oscillations, the term Atlantic Multidecadal Variability (AMV) is used in this study, as suggested by Cassou et al. (2018). Sutton and Hodson (2005) and Knight et al. (2006) highlighted the influence of the AMV on temperature and precipitation during the summer season over Europe, although the signal was most pronounced in Northwestern Europe. More recently, Sutton and Dong (2012) showed that the rapid warming over Europe from the 1990s onwards was directly linked to the overall warming pattern of the North Atlantic, hence the positive phase of the AMV. Changes in precipitation were found to be linked to changes in atmospheric circulation via the AMV by Ghosh et al. (2017). They showed that patterns of atmospheric circulation variability over Europe (and thus precipitation variability) are closely linked to Atlantic Sea Surface Temperature (SST) patterns reflecting the multidecadal AMV pattern. In addition, using hydrometeorological reconstructions, Bonnet et al. (2017) highlighted multidecadal changes in snow cover over the French Alps at the end of spring relative to variations in spring precipitation which influence summer river flows. The impact of characteristic SST patterns on the preferred location of the jet stream has also been documented by Brayshaw et al. (2011) and Baker et al. (2017). These studies highlight the importance of meridional SST gradients in shaping the strength and location of the mid-latitude westerlies and thus of the eddy-driven jet stream, and its influence on the atmospheric flow over Europe.

Overall, the literature mentioned above suggests changing average precipitation over northwestern Europe in reaction to altered multidecadal AMV conditions. However, the signal is less clear for other parts of Europe, such as the Alpine region. Moreover, the association of AMV with the interannual precipitation variability is not investigated at all notwithstanding its importance in the context of floods and droughts. This paper therefore analyses the variations of interannual summer precipitation variability in the Alpine region and its potential drivers with respect to atmospheric and oceanic modes of circulation on multidecadal time scales. The following science questions are addressed:

1. How did the interannual variability of summer precipitation in the European Alpine Region evolve between 1900 and 2018?
2. How are changes in the interannual variability of summer precipitation related to changes in the atmospheric circulation?
3. What is the role of the North Atlantic sea surface temperatures in driving changes in the interannual variability of summer precipitation on multidecadal time scales?

The paper is structured as follows: in Sect. 2 the datasets are introduced as well as the method for quantifying interannual variability and the estimation procedure of two circulation indices. The results Sect. 3 is divided into three parts. The first addresses the temporal evolution of interannual variability of summer precipitation in the GAR, the second assesses the drivers of these changes on regional and local scales, and the third deals with large scale atmospheric and oceanic drivers. In Sect. 4 the results are discussed in relation to the relevant literature and recommendations for further work are given.

## Data and methods

**Fig. 1:**
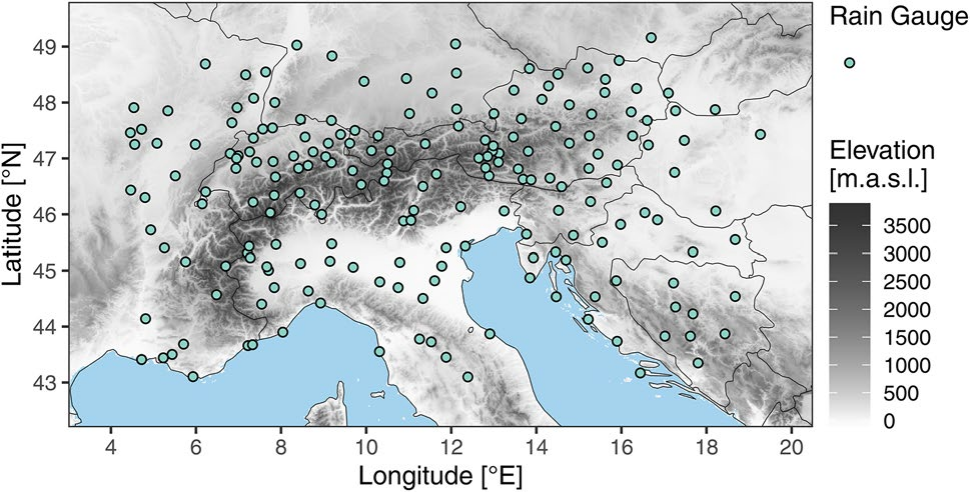
Map of the Greater Alpine Region (GAR) and location of HISTALP rain gauges

In this study precipitation data from the HISTALP database (Auer et al. 2007) is used, which consists of homogenized, high quality station data at a monthly time resolution covering the Greater Alpine Region (GAR, Fig. 1).

We focus on the period to 1900 to 2018 over which the station density does not change much. In the year 1900 for example 210 stations are available, which is 98% of the entire number of precipitation stations included in the HISTALP data base (in total 214). From the monthly time series we calculate seasonal means over the summer months (June, July, August), standardize each series to zero mean, and then average the series over the entire Alpine domain. From the average series we calculate the temporal standard deviation between years as:

$$ \sigma_{P } \left( i \right) = \sqrt {\frac{{\mathop \sum \nolimits_{j = i - m + 1}^{i} \left( {P_{j} - \overline{P}} \right)^{2} }}{m - 1}} $$

where $\sigma_{P}$ is the standard deviation of summer precipitation centered around year $i$ which is estimated from a time window of length $m$ (*i* ± 10 years), $P$ represents precipitation, $j$ is the index for the year within the sampling window and $\stackrel{-}{P}$ is the mean summer precipitation within the sampling window. At the beginning and the end of the time series the window is gradually reduced and therefore at the first and last year only 11 years are used for estimating ${\sigma }_{P}$, which introduces some uncertainty. For most of the analysis in this paper a 20-year window for moving averages and for identifying periods of interest is used. This choice is based on preliminary analyses which showed that this time window reveals most distinct differences between different time periods.

Additionally, we use the daily reconstructed circulation type (CT) classification of Schwander et al. (2017) covering the period from 1900 to 2009. It is based on the operationally used classification of MeteoSwiss (Weusthoff 2011) and consists of seven different circulation types (acronym: CAP7) tailored to the Alpine region using daily station data of mean sea level pressure. We extended the time series to 2018 by classifying the mean sea level pressure data from ERA-interim (Dee et al. 2011) using the COST733 classification software (Philipp et al. 2010). The application of ERA-interim data here is consistent with Schwander et al. (2017) who have used ERA40 and ERA-interim for calibrating their reconstruction. The skill of the classification and its ability to explain the meteorological drought evolution in the GAR have been shown by Schiemann and Frei (2010) and Haslinger et al. (2019), respectively.

**Fig. 2:**
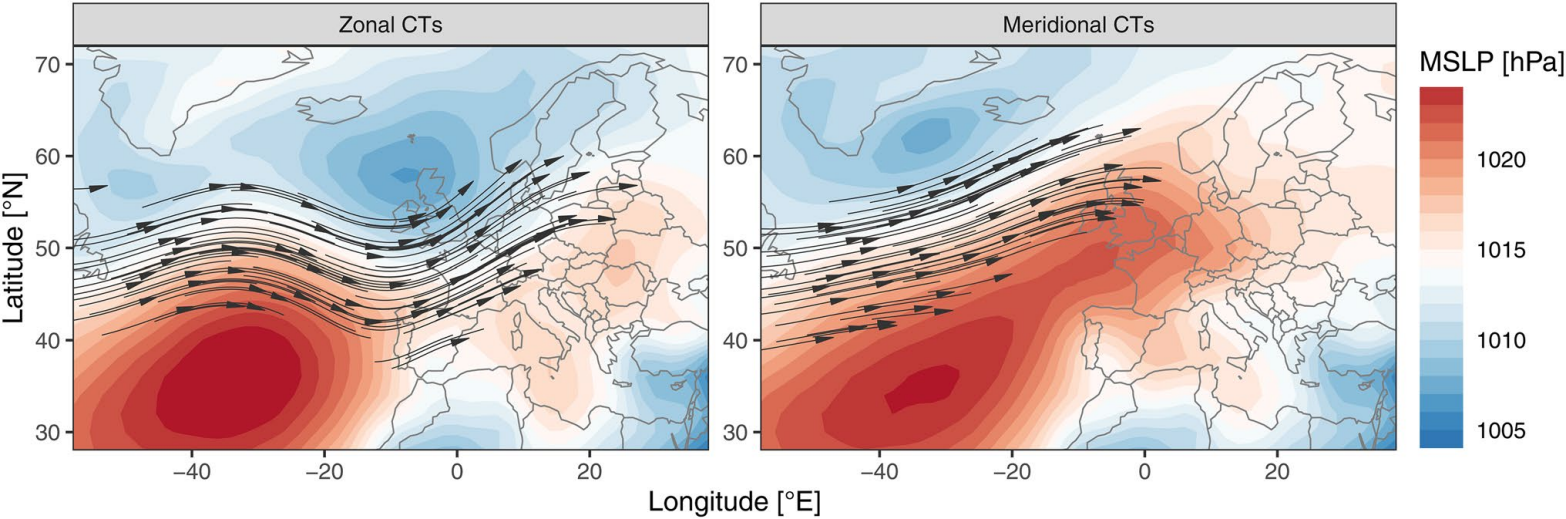
Average (1981–2010) summer Mean Sea Level Pressure (shading) for Meridional (left panel) and Zonal (right panel) CTs during; Arrows indicate the dominant flow at 500 hPa

From the daily circulation types, a Meridional Flow Index MFI representing the dominant flow regime (Zonal versus Meridional Flow), is calculated for each summer as

$$ MFI_{i} = \left( {\frac{{M_{i} }}{{\overline{M}}}} \right) - \left( {\frac{{Z_{i} }}{{\overline{Z}}}} \right) $$

where $M$ and $Z$ are the frequencies of meridional and zonal circulation types in (the summer of) year $i$, respectively and $\overline{M}$ and $\overline{Z}$ are the corresponding means over the period 1900–2018. An MFI larger than zero indicates more days with meridional than with zonal flow compared to the long term mean, while the opposite is the case for MFI smaller than zero. Zonal circulation types usually have a strong westerly flow component and large horizontal pressure gradients, respectively. Here we consider type 2 (West-Southwest, cyclonic, flat pressure) and type 3 (Westerly flow over Northern Europe) of the Schwander et al. (2017) classification to represent zonal circulation types. Meridional circulation types have a stronger northerly and easterly flow component and smaller pressure gradients over the whole domain. We consider type 1 (Northeast, indifferent), type 4 (East, indifferent) and type 6 (North cyclonic) to represent meridional circulation types. Figure 2 shows the average summer sea level pressure pattern and the related prevalent upper level flow of the combined meridional and zonal CTs, respectively.

In addition to the MFI a measure of persistency (PER) of the CTs is introduced. We follow the approach of Richardson et al. (2019) who used a Markov Chain to model persistency of weather patterns. They used different levels of complexity of their model, from simple, first order to higher order Markov Chains. However, Jordan and Talkner (2000) noted that a first-order model may be sufficient for describing the main stochastic characteristics of the weather type sequences. The assumption of first-order Markov Chains is that the probability for a CT to occur is solely dependent on the CT of the previous day. The transition probabilities are given by the transition matrix with its elements $p_{i,j} = {\text{Pr}}(CT_{t} = i|CT_{t - 1} = j)$, for $i, j = 1, \ldots , 7$. From this transition matrix we calculate the persistency index PER by:

$$ PER = \frac{1}{n}\mathop \sum \limits_{i = 1}^{n} p_{i,j} $$

which is the average transition probability of a subset of $$ p_{i,j}$$ with same-state (from one CT to the same CT) transitions. A high average same-state transition probability across all CTs lead to a high value of PER, indicating persistence.

In order to analyze the potential coupling between soil moisture and precipitation amount at a daily scale we use daily gridded meteorological fields from the E-OBS database (Cornes et al. 2018) for the period 1950–2018. Specifically, we use the daily precipitation fields as well as the Climatic Water Balance (CWB, precipitation minus potential evapotranspiration) over a 90 day (right sided) window, averaged over the GAR as a large scale proxy for soil moisture conditions (Mueller and Seneviratne 2012; Whan et al. 2015; Herold et al. 2016). Potential evapotranspiration is calculated by the Hargreaves equation (Hargreaves 1975; Hargreaves and Allen 2003) from the E-OBS minimum and maximum daily air temperature fields. Daily precipitation anomalies are calculated for the summer season with respect to the 1951–2010 summer daily mean precipitation sum.

In addition North Atlantic sea surface temperature anomalies from the ERSST V5 dataset (Huang et al. 2015) and mean sea level pressure and zonal wind speed from the 20th Century Reanalysis V2c (Compo et al. 2011) are used. From the mean sea level pressure fields the NEW (North-Atlantic-European East West) mode (Ghosh et al. 2017) is calculated by a Principal Component analysis of 10-year moving average summer mean sea level pressure fields over the domain 40° N–80° N and 60° W–100° E. Following Ghosh et al. (2017), the first Principal Component is denoted the NEW-mode.

## Results

### Temporal evolution of ${{\varvec{\sigma}}}_{{\varvec{P}}}$

**Fig. 3:**
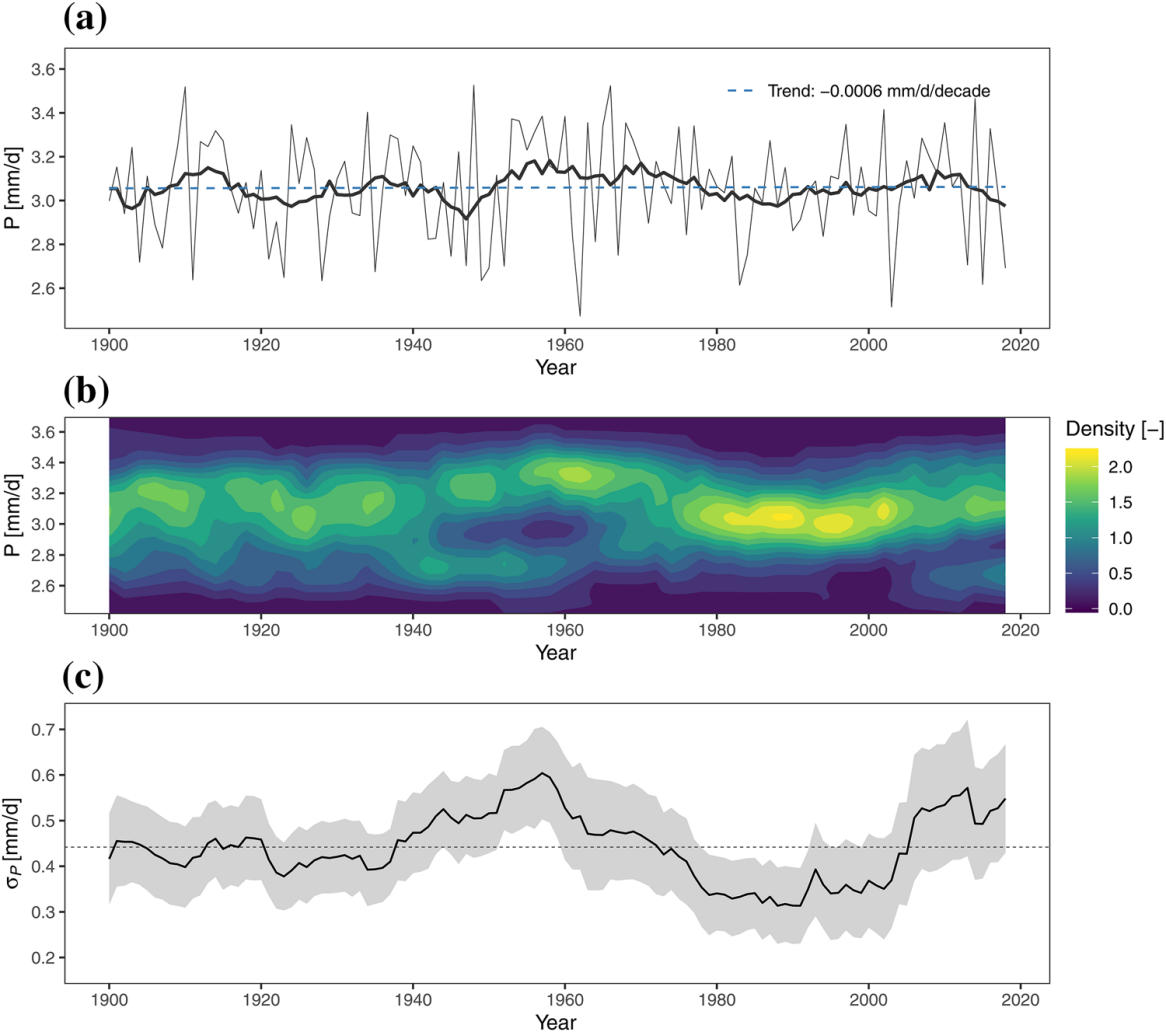
**a** Time series of average daily summer precipitation in the GAR (thin line), 20-year moving average (thick line) and linear trend (dashed blue line); **b** 20-year moving probability density of average daily summer precipitation (using a Gaussian Kernel estimate of the each 20-year sample); **c** 20-year running standard deviation of summer precipitation ${\sigma }_{P}$ with ± 1 standard deviation error band (grey) derived from bootstrapping, the dashed line indicates the mean ${\sigma }_{P}$ over the 1901–2018 period

Summer precipitation in the GAR shows essentially no linear trend over the period 1900 to 2018 (Fig. 3a). The Sen’s Slope estimate (Sen 1968) is − 0.0006 mm/decade (p = 0.92), meaning a linear trend model is not justified. These findings are consistent with the lack of trend in GAR summer precipitation (Brunetti et al. 2006), but who found pronounced variability on decadal time scales. This behavior is also evident Fig. 3a (20-year moving average), revealing wetter conditions during the 1910s and 1950s as well as dryer summers particularly in the 1940s.

The moving 20-year kernel-estimate of average summer precipitation (Fig. 3b) clearly shows a change of the shape of the distribution. Most of the time the shape is unimodal, but bimodal during the middle of the twentieth century and at the beginning of the twenty-first century. Accordingly, the Dip Test of Unimodality (Hartigan and Hartigan 1985) gives significant deviations (p-value < 0.05) from an unimodal distribution of the average summer precipitation from 1941–1962. Suggested bimodality at the beginning of the twenty-first century is not quite significant (p-value 0.27), although this might be caused by the fact that ${\sigma }_{P}$ is still negative during the first years of the twenty-first century. The changes in modality point toward regime changes caused by precipitation generating mechanisms in the GAR.

Changes in the shape of the distribution coincide with changes in the interannual variability of summer precipitation. Figure 3c shows the 20-year moving window standard deviation ${\sigma }_{P}$, the grey band indicates the ± 1 σ error. From the 1940s onwards ${\sigma }_{P}$ is characterized by multidecadal variations, where the time periods 1947–1966 (P1) and 1999–2018 (P3) show large ${\sigma }_{P}$, and the period 1975–1994 (P2) shows extremely low ${\sigma }_{P}$ compared to the rest of the time series. The differences in variance of P1 and P2 are significant at the 95% level (p-value of the F-statistic: 0.03) and those of P2 and P3 at the 90% level (p-value of the F-statistic: 0.07).

### Regional and local scale drivers

**Fig. 4:**
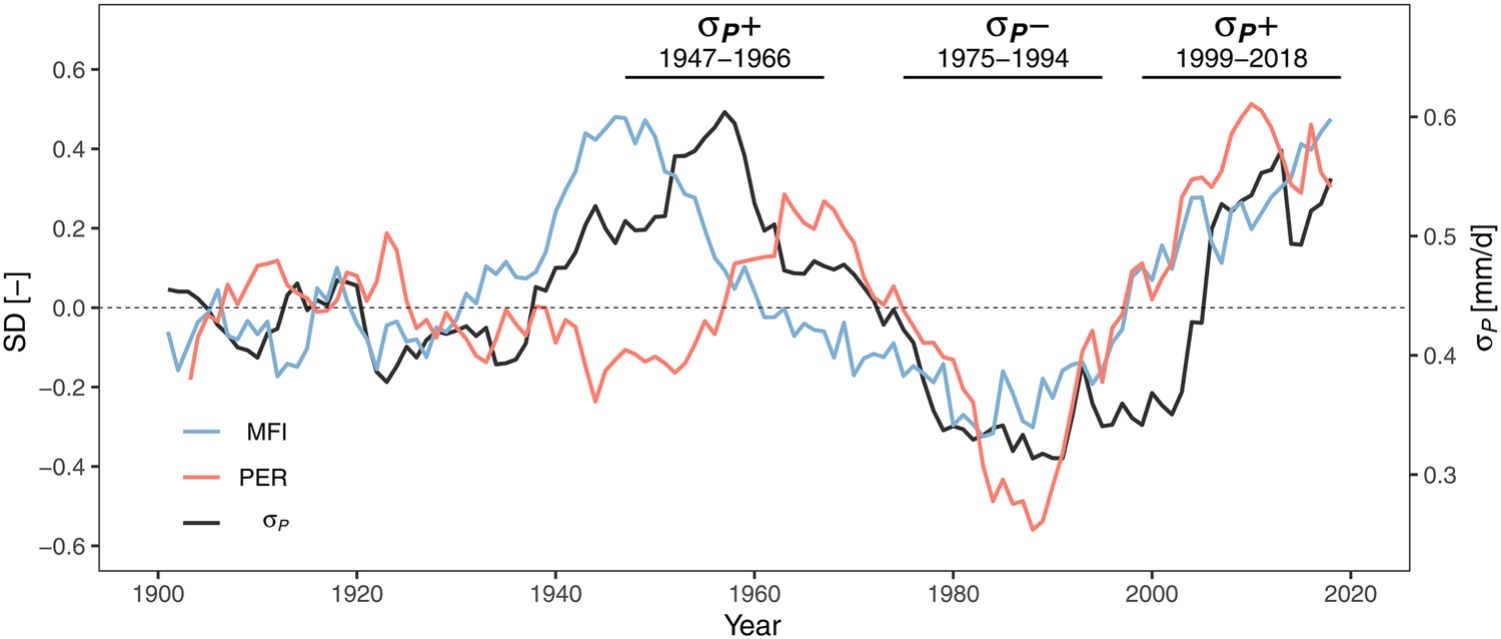
20-year moving averages of standardized Meridional Flow Index (MFI) (blue) and persistency index (PER) (red) time series and interannual variability of summer precipitation ${\sigma }_{P}$ (black, same as in Fig. 3c) highlighted are 20-year periods with particularly high (${\sigma }_{P}$ + 1947–1966 and 1999–2018) and low (${\sigma }_{P}-$ 1975–1994) interannual variability; the dashed line indicates the zero-line for the standardized indicators MFI and PER and the mean of ${\sigma }_{P}$ for the period 1901–2018

First of all, the atmospheric circulation as a primary driver for precipitation variability is considered, using the MFI and PER as indicators of the general flow regime in terms of direction and persistency (Fig. 4). During the beginning of the twentieth century until 1930 the MFI is always close to average conditions. From the 1930s the MFI dynamics increase. It reaches highly positive values in the 1940s and 1950s (indicating enhanced meridional flow), negative anomalies around the 1980s (indicating enhanced zonal flow), followed by a quick rise until the end of the time series. Apart from some temporal offsets (the positive peak of the MFI is earlier than that of ${\sigma }_{P}$), the MFI shows very similar multidecadal dynamics as those of ${\sigma }_{P}$, with a spearman rank correlation 0.69.

To some extent similar temporal behavior is also apparent in the PER time series. Similar to the MFI and ${\sigma }_{P}$, the PER shows only marginal dynamics until 1940, followed by a slightly negative phase in the 1940s and positive phase during the 1960s. In the 1980s, the PER reaches minimum values followed by a steep increase afterwards. The spearman correlation of ${\sigma }_{P}$ and PER is slightly lower with 0.42.

The decadal variability of the persistency of CTs (PER in Fig. 4) is however not as easy to interpret in terms of large scale modes of circulation. The steep rise in persistency from the 1980s onwards is often attributed to recent climate change and the mechanism of Arctic Amplification of surface air temperature change, which slows down the Rossby wave propagation through reduced latitudinal temperature gradients (Francis and Vavrus 2012; Coumou et al. 2015). However, there is a secondary peak in persistency in the late 1950s and the 1960s. Additional analyses, not shown here, suggest that this peak could be potentially attributed to a positive phase of the Scandinavian Pattern (Comas-Bru and Hernández 2018) related to blocking over Scandinavia. Hofstätter and Blöschl (2019) showed that Scandinavian blocking is often associated with negative North Atlantic Oscillation (NAO) and Arctic Oscillation (AO) conditions. These in turn have the potential to trigger cyclogenesis in the Western Mediterranean and persistent cyclonic circulation which frequently causes heavy rain in the Alpine region with potential for large scale flooding (Hofstätter et al. 2018).

**Fig. 5:**
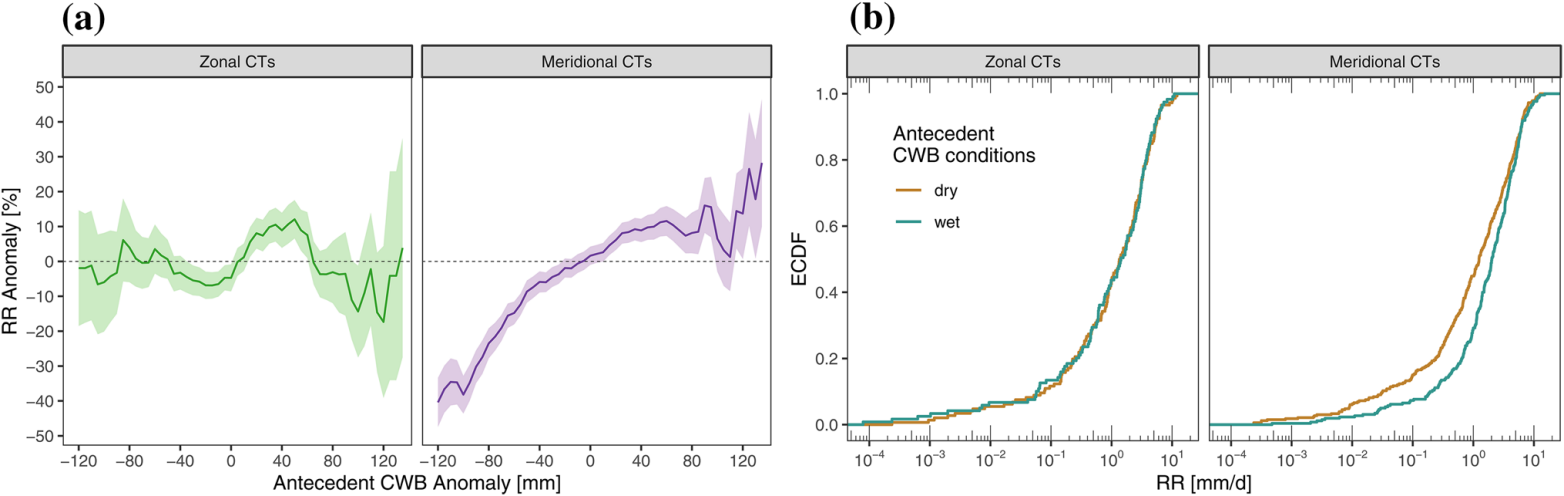
**a** E-OBS daily precipitation (RR) anomalies stratified by Circulation types (Meridional CTs: purple, Zonal CTs: green) and plotted against antecedent, until the day before, anomalies of the Climatic Water Balance (CWB) of the preceding 90 days. Shaded bands indicate ± 1 standard error of the mean precipitation anomaly estimation over a moving window of 50 mm CWB, **b** Empirical Cumulative Distribution Function (ECDF) of daily precipitation totals stratified by CTs (Meridional CTs: right, Zonal CTs: left) and antecedent moisture conditions expressed by below the 10th (above the 90th) percentile of the CWB for dry and wet conditions in brown and blue, respectively

In a study on atmospheric drought processes in the GAR (Haslinger et al. 2019) found that the distribution of precipitation anomalies during dry soil moisture conditions differed significantly from those during wet soil moisture conditions. They also found that these difference were particularly pronounced for meridional CTs and interpreted these findings as a result of positive soil moisture-precipitation feedbacks and self-enforcing drought conditions under prevailing meridional flow (MFI > 0) if the antecedent soil moisture conditions are already dry. Figure 5a presents a similar analysis, considering the whole distribution of antecedent moisture conditions in relation to precipitation. For days with zonal CTs (MFI−), the precipitation anomalies range about − 10 and + 10% around the mean of 2.40 mm/day, with rather large standard error ranges for very wet conditions (antecedent CWB anomaly > 100 mm), so a clear dependence on antecedent soil moisture cannot be seen in this case. For days with meridional CTs (MFI+), on the other hand, there is a clear relationship to observed precipitation. During extremely dry conditions (antecedent CWB anomaly < − 100 mm) precipitation is typically 40% lower than the mean of 2.75 mm/day. During very wet conditions (antecedent CWB anomaly >  + 100 mm) the precipitation anomalies reach up to + 20%.

The dependence of precipitation anomalies on antecedent CWB anomalies for the meridional CTs supports the existence of a soil moisture-precipitation feedback. In contrast, for zonal CTs, i.e. during periods of enhanced zonal flow, large scale moisture transport into the region may be larger and thus the impact of antecedent soil moisture on precipitation smaller.

Under zonal flow (Fig. 5b, left), the distribution function of daily precipitation is very similar on days with dry and wet soil moisture conditions, and the Kolmogorov–Smirnov-Test indicates no significant difference (p-value 0.92). In contrast, under meridional flow (Fig. 5b, right), the distributions are rather different. When the soils are dry, there is a much higher probability of non-exceedance of daily precipitation of a given magnitude to occur than when the soils are wet, and the Kolmogorov–Smirnov-Test indicates highly significant differences (p-value < 0.001). This result supports the interpretation that the atmospheric flow regime in the Greater Alpine Region may trigger or suppress local scale feedbacks of precipitation with soil moisture.

### Large scale atmospheric and oceanic drivers

**Fig. 6:**
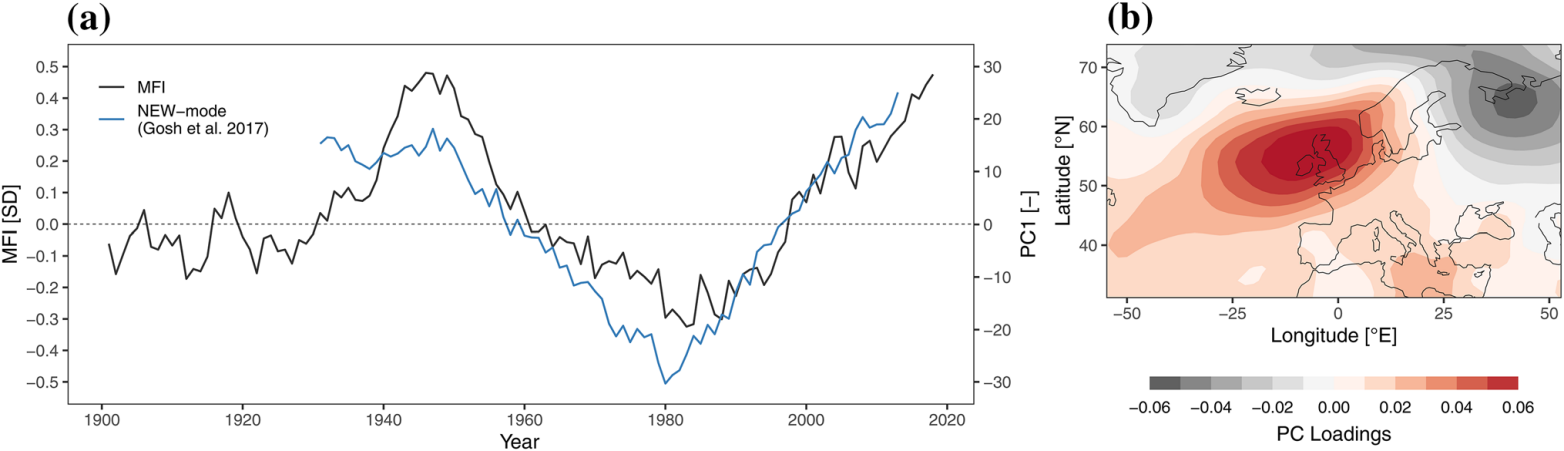
**a** Time series of the standardized Meridional Flow Index (MFI) (10-year moving average, black line) and the North-Atlantic-European East West (NEW)-mode (blue line), **b** corresponding loading pattern of the first principal component of mean sea level pressure in the domain 100° W–80° E and 30° N–80° N as defined by (Ghosh et al. 2017)

In this context it is of interest to explore the relationship between the regional scale circulation and the modes of circulation over the wider North Atlantic-European Domain. In general, the North Atlantic Oscillation (NAO) is the primary large scale circulation pattern, which is the case on monthly or annual-, but to a less extent on multidecadal time scales. Figure 6a presents the time series of the 10-year moving averaged MFI and the NEW-mode (Ghosh et al. 2017). The positive phases of the NEW-mode during the 1940s and 1950s are due to positive mean sea level pressure anomalies over the British Isles and negative anomalies over Western Russia as shown in the loading pattern of Fig. 6b. The opposite is the case for the negative phase in the early 1980s. Overall, the multidecadal variability of the NEW mode is closely aligned with that of MFI, with a spearman rank correlation of 0.86 (Fig. 6a), suggesting a tight relation between regional flow regimes in the Alps embedded in a general large-scale mode of circulation.

**Fig. 7:**
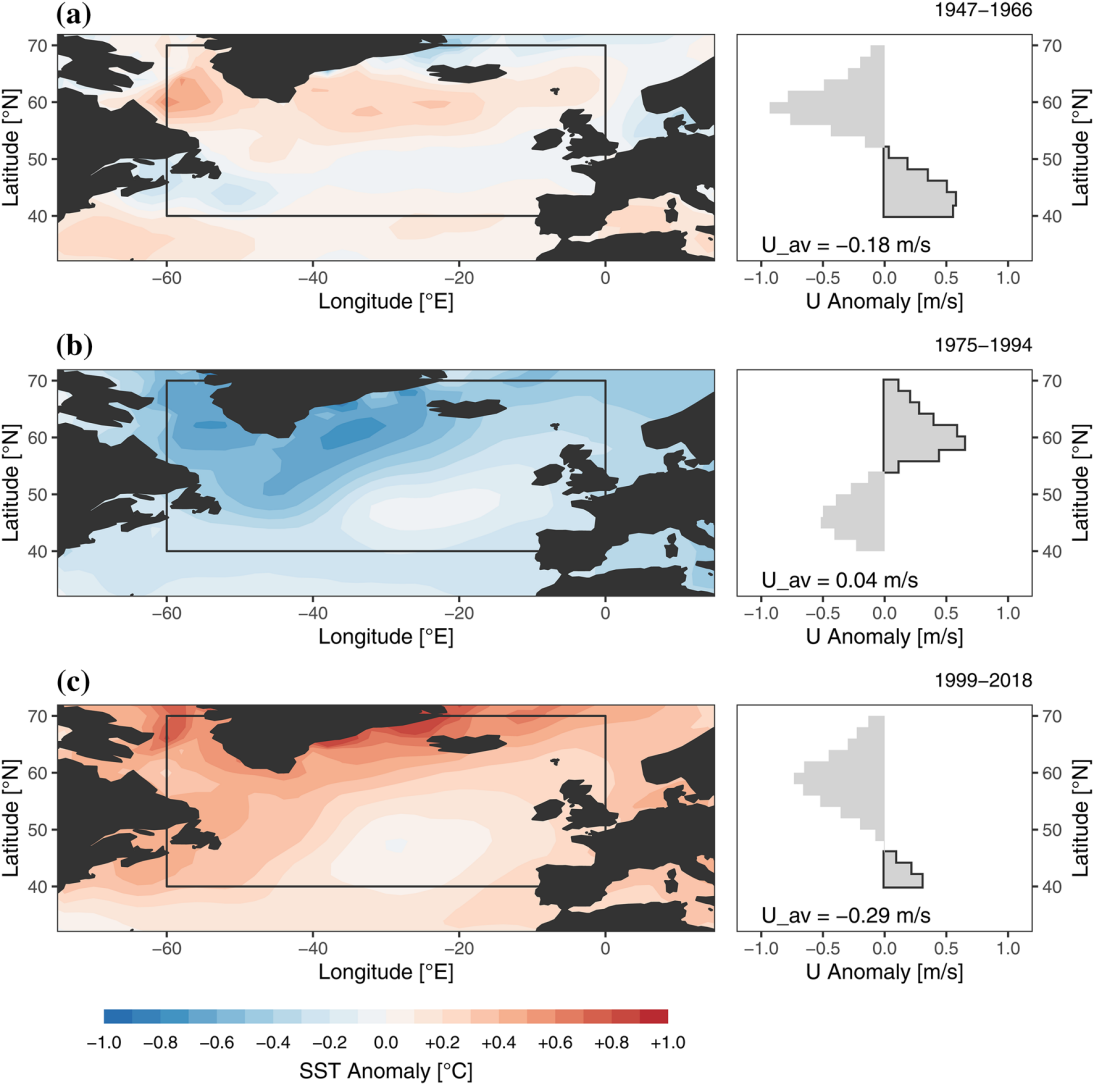
Sea surface temperature (SST) anomalies (left panels) and zonal 850 hPa wind speed (U) anomalies (reference period 1901–2010) averaged over latitudinal bands (right panels) for the 3 periods of significant ${\sigma }_{P}$ deviation as in Fig. 4: **a** P1(1947–1966), **b** P2 (1975–1994) and **c** P3 (1999–2018), the average U anomaly of the latitudinal range from 40° to 70° North is indicated at the bottom of each plot (U_av)

Ghosh et al. (2017) proposed distinct SST patterns in the Atlantic as a main driver for the NEW-mode. They suggested that positive heat flux anomalies in the northwestern Atlantic induce a negative pressure anomaly east of the heat source up to 500 hPa, thus generating an east–west wave-like pressure anomaly. In order to shed light on these processes Fig. 7 presents the SST anomalies for the periods of distinct ${\sigma }_{P}$ anomalies (c.f. Fig. 4). Large differences in the spatial distribution of warm and cool SST anomalies are apparent, particularly when period P2 is compared to P1 and P3. P1 and P3 show positive SST anomalies most pronounced around 60° N (the area under investigation is indicated by the black rectangle ranging from 40 to 70° N) and negative anomalies around the region of 50° N. During these periods most of the North Atlantic shows positive SST anomalies. On the other hand, P2 shows the opposite pattern, with generally negative SST anomalies over the whole North Atlantic and particularly north of 50° N.

Regardless of the drivers of SST pattern variability, the implications for the atmospheric circulation is shown in numerous studies. For example Brayshaw et al. (2011) and Baker et al. (2017) showed that the location and the speed of the polar jet stream is strongly determined by the strength of the meridional surface temperature gradient. In the present study we use the zonal wind anomalies at 850 hPa as an indicator for the eddy driven jet following the approach of Woollings et al. (2014). We choose the domain from – 60 to 0° W and 40–70° N which is similar to that of Woollings et al. (2014), however we constrained the latitudinal extent, since most variability of the eddy driven jet appears within this range (c.f. Fig. 4 in Woollings et al. 2014). In the right panels of Fig. 7a–c the zonal wind speed (U) anomalies (850 hPa) averaged for latitudinal bands are displayed. During periods P1 and P3 positive U anomalies occur in the southern part of the mid-latitude domain, while in P2 they occur in the northern part. In period P2, the average U anomaly is positive (+ 0.04 m/s) while in P1 and P3 it is negative (− 0.18 and − 0.29 m/s respectively). These patterns imply changes in the strength of the westerly airflow and meridional shifts of the eddy driven jet as found by others as well (e.g. Woollings et al. 2014). Figure 7b suggests that during the negative ${\sigma }_{P}$ phase (P2) the jet stream shift was likely driven by the negative SST gradient, which enhanced the meridional surface temperature gradient and thus strengthened the jet and shifted it towards the North. The area with most pronounced U anomalies concurs with negative SST anomalies and large SST anomaly gradients southwards. During the positive ${\sigma }_{P}$ phases (P1 and P3), the meridional SST anomaly gradient is positive towards the North, hence resulting in an overall weaker absolute SST difference from South to North. The jet stream is shifted towards the south during both periods and again concurs with the regions of negative SST anomalies and large SST anomaly gradients southwards.

**Fig. 8:**
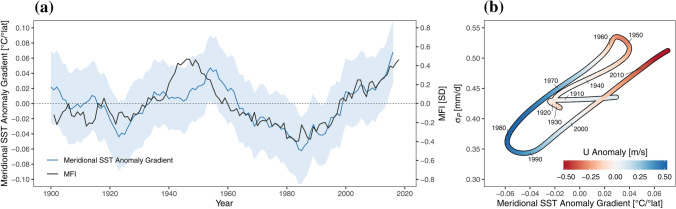
**a** 20-year moving averages of the standardized Meridional Flow Index (MFI, black) and the meridional sea surface temperature (SST) Anomaly Gradient (blue) for the area 60–0° W and 40–70° N. The blue band indicates the ± 1 standard deviation. **b** Phase diagram of the relationship between the Meridional SST Anomaly Gradient and the interannual variability of summer precipitation ${\sigma }_{P}$; the anomaly of the U wind component is shown as color shading. All time series are filtered using a Gaussian filter with a 1 σ width of 13 years

Using an idealized Global Climate Model setup, Baker et al. (2017) showed that the storm tracks over the Euro-Atlantic sector, and hence the synoptic situation downstream, are strongly linked to the location of the jet stream. This is consistent with the alignment of periods of large ${\sigma }_{P}$ anomalies with those of MFI, U and SST pattern anomalies in the mid-latitude North Atlantic found here. To illustrate the related importance of SST gradients, the meridional SST anomaly gradient is displayed in Fig. 8a along with the MFI. Positive gradients denote a situation when the temperatures in the north of the domain were warmer than usual and those in the south were colder than usual. The consistency of the SST gradient over the study period and the atmospheric flow regime over the Alpine region (as quantified by the MFI) is striking. Over the entire period the correlation coefficient between these two variables is 0.77, for 1961–2016 it is 0.90. The phase diagram of ${\sigma }_{P}$ and the meridional SST anomaly gradient (with U anomalies in colours) in Fig. 8b shows a striking consistency of all three variables. This close relationship, however, only establishes itself from around 1920 onwards. During the first two decades of the twentieth century ${\sigma }_{P}$, MFI and PER (Fig. 4) and the meridional SST anomaly gradient (Fig. 7a) do not vary much. This behavior could be related to a different jet stream configuration across the Euro-Atlantic sector. Woollings et al. (2014) showed that jet latitude and the jet speed varied considerably during the twentieth century and that there was a likely step change in summer jet speed around 1920 with an increase of around 1 m/s (Fig. 8c in Woollings et al. 2014). However, it is more likely that uncertainties in the underlying datasets cause uncertainties in the given analysis prior to 1930. Several studies point towards growing uncertainties in reanalysis data beyond 1930, e.g. Gastineau and Frankignoul (2015) and Krueger et al. (2013). Moreover, Ghosh et al. (2017) used only data from 1930 onwards from the 20CR dataset, because of unrealistic response patterns of the atmosphere considering diabatic heating on the North Atlantic during the early decades of the twentieth century.

**Fig. 9:**
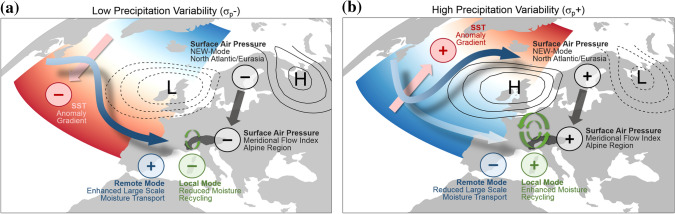
Schematic processes related to low (**a**) and high (**b**) interannual variability; two contrasting “modes” driving interannual variability in the Alpine region are distinguished: (i) the Remote Mode which is in its positive phase **a** if there is enhanced moisture transport from the Atlantic ocean (blue arrow) and (ii) the Local Mode which is in its positive phase **b** if there is enhanced moisture recycling due to soil moisture-precipitation feedbacks (green circular arrows) in the Alpine region and vice versa; these modes are driven by regional (Meridional Flow Index) atmospheric circulation characteristics embedded in large scale pressure distributions (NEW-mode) which is related to North Atlantic SST Anomaly Gradients in the mid-latitudes as depicted by the shades area and the light red arrows

Figure 9 summarizes in a conceptual framework the main drivers of the variations of interannual precipitation variability across scales highlighted in the previous sections. The framework distinguishes between two main states. During a state of low precipitation variability MFI is negative (Fig. 9a), and is associated with dominant zonal CTs carrying moisture predominantly from the Atlantic to the Alpine region. These regional circulation characteristics are steered by large scale atmospheric variability modes on multidecadal time scales. A negative MFI is associated with a negative NEW-mode (NEW–), with a dominant low pressure system over Northwestern Europe. Moreover, the meridional distribution of SST anomalies in the mid-latitude Atlantic acts as a precursor for the atmospheric circulation by strengthening the westerly air flow in case of a negative meridional SST anomaly gradient (positive anomaly southwards and negative anomalies northwards). The crucial part of the process chain is the atmospheric circulation and the prevailing moisture flow over the Alpine region, which could be termed to be in “Remote Mode” when MFI is negative, due to enhanced horizontal moisture transport.

During a state of high precipitation variability (Fig. 9b, positive MFI) on the other hand, the opposite is the case and large scale modes of variability have an opposite sign (positive SST anomaly gradient, positive NEW-mode). In the Alpine region the vertical moisture flow is enhanced due to higher local moisture recycling which is a result of less zonal CTs and thus more meridional/weak pressure gradient CTs. Under these conditions, the “Local Mode”, it is more likely that positive soil moisture-precipitation feedbacks are triggered, which could push to even wetter or dryer conditions during the summer, depending on the antecedent soil moisture conditions and thus drive higher interannual precipitation variability.

## Discussion

Summer precipitation totals in the Alpine Region do not exhibit a systematic trend over the period 1900–2018. However, we find significant low frequency periodicity of interannual variability. Wetter conditions prevail during the 1910s and 1950s on one hand, dryer summers are particularly occurring in the 1940s and most recently on the other hand. For most of the study period, summer precipitation variability is linked to a uniform distribution. During the 1941–1962 period, however, we find a significant bimodality regime, which is in perfect alignment with an enhanced interannual variability of summer precipitation. Most important, the beginning of a similar bimodal phase is suggested from around 2005. Phases of enhanced interannual variability occur in synchronization with a dominant two-phase state of the atmospheric circulation over the Alps. These modes are driven by regional atmospheric circulation characteristics (depicted by MFI variations) embedded in large scale pressure distributions (NEW-mode). During NEW− (NEW+) a dominant low (high) pressure system over Northwestern Europe (Western Russia) is observed. Findings suggest that enhanced meridional flow (local mode) increases precipitation variability through positive soil moisture precipitation feedbacks on the regional scale, whereas enhanced zonal flow results in less variability through constant moisture flow from the Atlantic and suppressed soil-moisture feedbacks with the land surface (remote mode). While these feedbacks tend to be hard to verify (Koster et al. 2017) and existing studies are mostly located in the subtropics, rather than in the mid-latitudes (Findell et al. 2011; Guillod et al. 2015) they do confirm the existence of such feedbacks. Moreover, there is one line of evidence also for the Alpine region. Sodemann and Zubler (2009) investigated moisture sources for Alpine precipitation for the period 1995–2002 and found that recycling ratios show rather large interannual variability and hence a strong dependence on atmospheric circulation, with clearly lower moisture recycling under zonal flow.

Furthermore the dominant state of the atmospheric circulation over the Alps in these periods appears to be steered by meridional sea surface temperature gradients in the North Atlantic acting on the latitudinal distribution and average zonal wind speed in the mid-latitudes. The SST patterns shown in Fig. 7 lead to the question if SST patterns are forced by the atmosphere (and at which time scale) or vice versa. While some earlier studies suggest that the atmosphere is the main driver for North Atlantic SST variability (Häkkinen et al. 2011), more recent studies emphasize the effect of SST anomalies on the atmosphere (Gastineau and Frankignoul 2015). McCarthy et al. (2015) and Routson et al. (2019) point out that, while the atmosphere is a primary forcing for North Atlantic SST variability on annual time scales, the opposite is the case on multidecadal and longer time scales. Two main processes seem to play an important role in the multidecadal variability of the North Atlantic SST (Frankcombe et al. 2010). One is a thermal Rossby wave mechanism inducing westward propagating temperature anomalies in the ocean leading to zonal and meridional temperature differences on a 20 year time scale (Frankcombe et al. 2008). Another mechanism relates to saline Rossby modes in the Arctic with southward propagating salt exchange at 70°North (Frankcombe and Dijkstra 2011).

Given the importance of North Atlantic SST patterns in modulating atmospheric flow it is pertinent to discuss these with respect to the Atlantic Multidecadal Variability (AMV, Enfield et al. 2001). The AMV expresses the average basin-wide SST anomalies in the North Atlantic and is a main driver for low frequency climate variability in the Northern hemisphere. The influence of the AMV on the European climate, particularly during summer, is well documented (Sutton and Hodson 2005; Knight et al. 2006; Sutton and Dong 2012). Although the impact on temperature is more pronounced and more extensive in space, some studies (Knight et al. 2006; Ghosh et al. 2017; O’Reilly et al. 2017) did find a signal of precipitation variations to be aligned with variations of the AMV in northwestern Europe, however, for the Alpine region the signal is less clear.

**Fig. 10:**
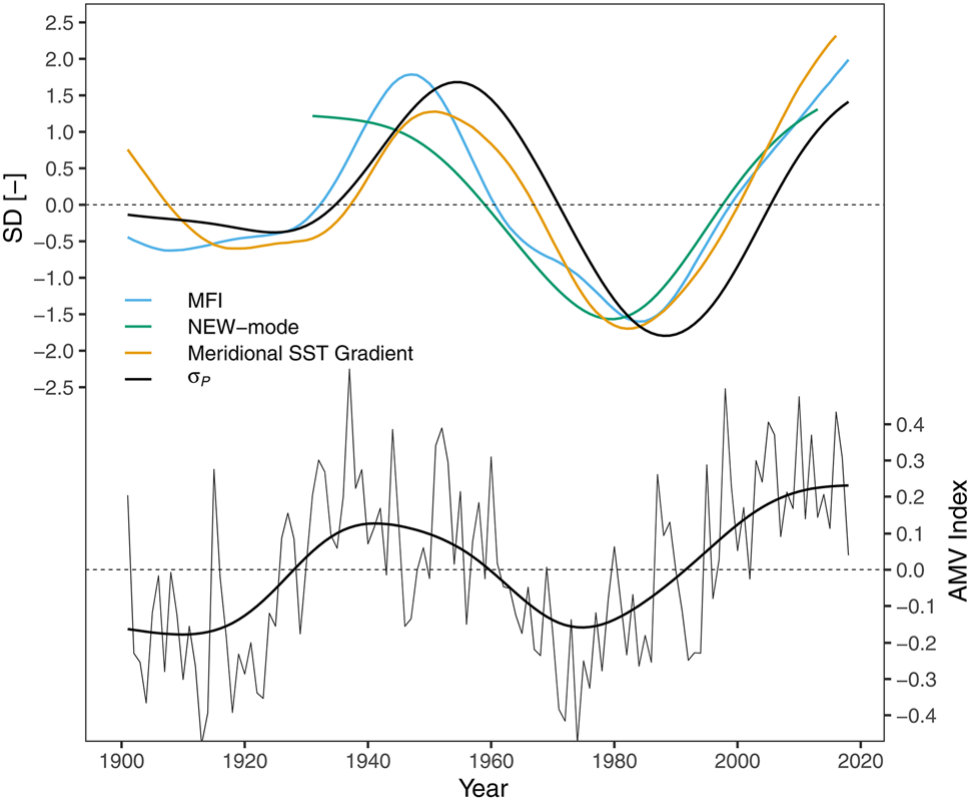
Upper panel: standardized and low-pass filtered time series of the Meridional Flow Index (MFI, blue), the NEW-mode (green), the Meridional Sea Surface Temperature (SST) Gradient and ${\sigma }_{P}$ using a Gaussian filter with a filter width of 50 years for 3σ, lower panel: Atlantic Multidecadal Oscillation (AMV) Index, annual values (thin line) and Gaussian filter with a filter width of 50 years for 3σ (thick line)

Figure 10 shows the low pass filtered time series of the AMV along with ${\sigma }_{P}$ and the other atmospheric and oceanic modes of circulation analyzed in this paper. All these modes and ${\sigma }_{P}$ seem to inherit the same low frequency pattern. Analysis of the red noise spectra using the method of Schulz and Mudelsee (2002) of ${\sigma }_{P}$ and the atmospheric and oceanic modes of circulation reveals significant (95% level) oscillations at a time scale of ~ 50 years for both the AMV and ${\sigma }_{P}$. The other modes do show some higher power of frequencies between 50 and 100 years, although not significant. Interestingly, during the first negative phase of the AMV (~ 1900–1925) ${\sigma }_{P}$ and the other atmospheric indices (MFI and NEW-mode) do not show a significant negative deviation. This might be related to relatively moderate signals of the meridional SST gradient (c.f. Fig. 8a) during this period, indicating that a distinct AMV phase is not necessarily affecting SST gradients. However, while the time scales of variability of the AMV and ${\sigma }_{P}$ are similar, the signals are shifted in time. A crosscorrelation analysis gives a maximum correlation (0.49) at a lag of 17 years with the AMV leading ${\sigma }_{P}$. Lagged correlations are furthermore found with the sea level pressure index as defined by Sutton and Dong (2012) with the AMV leading 5–6 years during spring (Boé and Habets 2014). Most recently, Bonnet et al. (2020) found a significant lagged correlation with precipitation over the Seine basin with the AMV leading 10 years. However, it is yet not clear what physical mechanisms drive this shifted behavior as the processes driving the AMV itself are not fully understood. Some studies indicate that the AMV is subject to the strength of the meridional overturning in the North Atlantic basin (Knight et al. 2005; McCarthy et al. 2015), thus pointing towards internal variability as a driver, other studies show that external forcing such as aerosols (Booth et al. 2012) and solar forcing (Malik et al. 2018) may play a role. Recently, Qin et al. (2020) suggested that a combination of different factors including internal variability and external forcings could be the origin of the AMV.

## Conclusions

In this paper we analyze the interannual variability of summer precipitation in the European Alpine Region and potential driving mechanisms on multidecadal time scales. We show that the changes in variability are directly linked to changes in predominant synoptic patterns over the Alpine Region (zonal vs. meridional flow) and suggest that soil moisture-precipitation feedbacks play a crucial role in this respect. Hence there is a strong indication that atmospheric circulation is controlled by the distribution of SST anomalies on multidecadal time scales in alignment with findings from previous studies (Ghosh et al. 2017).

The results of this study however indicate that the processes driving SST patterns are in close relation with those of the atmosphere on multidecadal time scale, thus shaping summer precipitation variability in the European Alpine region. Given that the key findings here are based on descriptive statistics with an underlying conceptual model of the processes involved, it would be vital to further investigate these with coupled ocean/atmosphere Global Climate Models to evaluate their robustness.

The findings also highlight the importance of natural climate variability for explaining precipitation characteristics in the European Alpine Region in the context of global climate change. Given that the processes driving interannual precipitation variability identified here are not always captured well in current Global Climate Models (Kravtsov et al. 2018) there is a continuing need for in-depth engagement of climate modelling with the synoptic mechanisms of ocean/land–atmosphere feedbacks. Future work should therefore focus on the evaluation of the skill of climate models in simulating multidecadal variations of ocean/land–atmosphere interactions and their implications for regional climate change.

## Data Availability

The authors would like to acknowledge following data providers: CAP7 circulation type reconstructions are obtained from Mikhael Schwander upon personal request, HISTALP precipitation data accessed from Central Institute for Meteorology and Geodynamics (ZAMG) (http://www.zamg.ac.at/histalp/), EOBS data accessed from European Climate Assessment and Dataset (https://www.ecad.eu/download/ensembles/download.php), 20CR V2c, ERSST V5 and AMV data accessed from NOAA Earth System Research Laboratory (https://www.esrl.noaa.gov/psd/data/gridded/data.20thCReanV2c.html, https://www.esrl.noaa.gov/psd/data/gridded/data.noaa.ersst.v5.html, https://www.esrl.noaa.gov/psd/data/timeseries/AMO/).

## Code availability

All analysis is carried out using the R programming language, code is available from the corresponding author by personal request.
